# Use of X-ray irradiation for inactivation of *Aspergillus* in cannabis flower

**DOI:** 10.1371/journal.pone.0277649

**Published:** 2022-11-15

**Authors:** Stephen Frink, Olivera Marjanovic, Phoi Tran, Yun Wang, Weihong Guo, Noahie Encarnacion, Donelle Alcantara, Bahman Moezzi, Gordon Vrdoljak

**Affiliations:** 1 Cannabis Testing Laboratory Branch, California Department of Cannabis Control, Richmond, California, United States of America; 2 Food and Drug Laboratory Branch, California Department of Public Health, Richmond, California, United States of America; Leibniz-Institut fur Naturstoff-Forschung und Infektionsbiologie eV Hans-Knoll-Institut, GERMANY

## Abstract

California cannabis regulations require testing for four pathogenic species of *Aspergillus–A*. *niger*, *A*. *flavus*, *A*. *fumigatus* and *A*. *terreus* in cannabis flower and cannabis inhalable products. These four pathogenic species of *Aspergillus* are important human pathogens and their presence in cannabis flower and cannabis products may pose a threat to human health. In this study, we examined the potential of X-ray irradiation for inactivation of cannabis flower contaminated with any of the four pathogenic species of *Aspergillus*. We determined that X-ray irradiation at a dose of 2.5 kGy is capable of rendering *Aspergillus* cells non-viable at low (10^2^ spores/g dried flower), medium (10^3^ spores/g dried flower) and high (10^4^ spores/g dried flower) levels of inoculation. We also showed that X-ray treatment of cannabis flower did not significantly alter the cannabinoid or the terpene profiles of the flower samples. Therefore, X-ray irradiation may be a feasible method for *Aspergillus* decontamination of cannabis flower. More work is required to determine the consumer safety of irradiated cannabis flower and cannabis products.

## Introduction

Although cannabis remains at the federal level a Schedule I substance under the 1970 Controlled Substance Act, it has rapidly gained legalization status at the state level. As of July 2022, only three states remain without any public access to legalized cannabis programs [1, 2]. California first legalized cannabis for medicinal use in 1996 under the Compassionate Use Act (Prop 215), then for adult recreational use in 2016 (Prop 64) (https://cannabis.ca.gov/cannabis-legislation/). Testing rules for safe cannabis consumption were established in 2017 under the Medicinal and Adult-Use Cannabis Regulation and Safety Act (MAUCRSA). MAUCRSA mandates that all inhalable cannabis goods be tested for microbial contaminants: Shiga-toxin producing *Escherichia coli*, *Salmonella* spp. and four pathogenic *Aspergillus* species (*A*. *fumigatus*, *A*. *flavus*, *A*. *niger*, and *A*. *terreus*). Increasing legalization has also led to a shift in attitudes by both consumers/patients and medical care providers in support of cannabis as a legitimate medical therapy [3–6]. Because of its Schedule I status, the rigorous safety and efficacy tests and oversight from the federal Food and Drug Administration (FDA) cannot be applied to cannabis for medical use [6, 7].

*Cannabis sativa* L. plants with its condensed, highly-branched large inflorescences play hosts to number of microorganisms, including bacteria and fungi that could be harmful if ingested or inhaled [8]. Analyses of microorganisms of cannabis samples collected from dispensaries or from commercially licensed producers from California, Massachusetts, Netherlands, and Canada have all identified opportunistic fungal and bacterial pathogens. Thompson *et al*. found *Aspergillus fumigatus*, *Cryptococcus laurentii*, *Mucor circinelloides*, *Klebsiella pneumoniae*, *E*. *coli*, *Salmonella*, *Enterobacter*, *and Bacillus* from cannabis collected from various northern California dispensaries [9]. Similarly, McKernan *et al*. [10], Verweij *et al*. [11], and Shapiro *et al*. [12] detected toxigenic *Pennicillium* spp., *Aspergillus* spp., and *Cryptococcus* spp. from commercially procured cannabis. Punja *et al*. [13] cultured 34 fungal taxa by swabbing dried cannabis flowers from three licensed commercial producers in British Columbia over a two-year period; the most prevalent fungal species in their study was *Penicillium*, *Cladosporium*, *Botrytis*, *Aspergillus*, *Fusarium*, *Alternaria*, and *Talaromyces*. Smoking, vaping or inhaling contaminated cannabis can lead to life-threatening systemic fungal infections. An analysis of a large database of health insurance claims from 2016 found that cannabis users were 3.5 times more likely than non-cannabis users to have a fungal infection − 43% of these fungal infections in cannabis users were due to aspergillosis [14]. Aspergillosis is a disease caused by the common mold *Aspergillus* [15, 16]. There are over 180 species of *Aspergillus* species, with less than 40 known to cause any infections in humans [17]. *Aspergillus* spores are everywhere and healthy individuals breathe them in daily without any adverse health effects [16, 17]. Individuals who are the most susceptible to *Aspergillus* infections tend to be immunocompromised, such as, people with HIV infection, rheumatoid arthritis, diabetes mellitus, bone marrow transplants, solid organ transplant recipients, leukemia or those undergoing chemotherapy [18–26]. The mortality associated with invasive aspergillosis in liver transplant patients and those with serious illnesses ranges from 66% to 90% [18, 27]. Cannabis is taken for a wide variety of medical reasons ranging from pain management, chemotherapy induced nausea and vomiting, cancer, seizures, epilepsy, HIV/AIDS, to anxiety/traumatic stress [3, 4]. Ensuring the safety of medical cannabis is imperative for critically ill patients. The Canadian and Dutch governments have allowed gamma irradiation of cannabis flowers to remove microbial contaminants [28, 29]. Canada also allows X-ray irradiation and electron beam (e-beam) irradiation for ensuring microbial sterility in cannabis and cannabis products [28].

Microbial pathogens can be eliminated using many different decontamination and sterilization methods; however, the methods for decontaminating medicinal plant materials while still retaining their bioactive properties are few [30]. A good sterilization method must not significantly alter the content, composition of, and characteristics of biologically active substances, such as cannabinoids, essential oils, terpenoids, flavonoids, poly-phenol acids, saponins, and other secondary metabolites [29–31]. Decontamination methods involving heat and chemical reagents can reduce/alter aromatic and biologically active compounds of plants or it can leave behind toxic residues [29, 30, 32]. In one study, cannabis was sterilized via autoclaving, by plasma H_2_O_2_, or by ethylene oxide gas prior to administering the cannabis to an immunocompromised patient [24]. All three methods succeeded in killing off all mold (including *Aspergillus*) and bacteria, but each method had significantly reduced Δ^9^-THC levels from 12.6% (plasma) to 22.6% (autoclave)/ 26.6% (ethylene oxide) [24]. Ethylene oxide is an extremely toxic and carcinogenic compound and has been banned in Europe because of safety and environmental concerns and also banned in the USA for treating ground spices [32, 33].

Ionizing radiation has several advantages for sterilizing plants for medicinal use. It is considered a cold pasteurization technique as it minimally raises the temperature in the product being sterilized; Sádecká [31] in a review of irradiation of spices noted a 0.36°C increase in product temperature per 1 kGy dose. Irradiation dose is measured in grays (Gy) which is the unit of energy absorbed in J kg^-1^ of material [34]. There are no chemical residues left behind and it preserves the product quality and characteristics [30, 32]. Ionizing treatment of dried herbs, spices, and vegetable seasonings at doses below 30 kilo grays (kGy) was authorized in 1992 by the USA for microbial decontamination [32, 35]. The International Atomic Energy Agency (IAEA) in Vienna, the Food and Agriculture Organization (FAO) in Rome, and the World Health Organization (WHO) made a recommendation that “irradiation of any food commodity up to an overall average dose of 10 kGy presents no toxicological hazards” [35]. There are three sources of ionizing radiation used for sterilizing plants or food items for human consumption: gamma irradiation, electron beam irradiation and x-ray irradiation. Gamma irradiation uses gamma rays emitted by decaying radioisotopes (^60^Co or more rarely ^137^Cs) and has high penetrance of the products. Electron beam irradiation (e-beam) generates electrons from an electricity-powered accelerator machine. It delivers high dosages in a short period of time but has low penetrance. X-ray irradiation is also an accelerator-based radiation, but with high penetrance. Bremsstrahlung X-rays are emitted when accelerated electrons hit a heavy metal target (i.e. tungsten, tantalum, gold) and are converted into photons [30, 33, 36, 37].

Ionizing radiation inactivates microorganisms and other living cells either as a direct action of the radiation or indirectly with the radiolysis of cellular water to form highly damaging free radicals such as H_3_O^+^, OH^-^, singlet oxygen (^1^O_2_), hydroxy radical (HO^+^), superoxide anion (O2^-^), or hydrogen peroxide (H_2_O_2_) [30, 33, 36]. These free radicals then interact with cellular components such as proteins, lipids, and DNA causing irrevocable damage leading to cell death [30, 33, 36]. A study has found that *Aspergillus* spp. and other toxigenic fungi contamination in four types of medicinal plants were eliminated after 10 kGy of gamma irradiation [38]. Similarly, Jerushalmi *et al*. completely inactivated microbial contaminants in cannabis flowers using gamma irradiation (7.5–8.37 kGy) and beta irradiation (e-beam, 10.26 kGy) [39]. Hazekamp showed that dried cannabis flowers exposed to 10 kGy of gamma irradiation did not alter THC and CBD levels or terpene composition qualitatively but terpene content was reduced by 10–20% [29]. X-ray irradiation (2–4 kGy) was successfully used by both Mahmoud *et al*. [40] and Jeong *et al*. [34] to inactivate pathogenic bacteria (*E*. *coli* O157:H7, *Listeria monocytogenes*, *Salmonella enterica*, and *Shigella flexneri*) from fresh spinach and other postharvest fungal contaminants (*Botrytis cinerea*, *Rhizopus stolonifera*, and *Penicillium expansum*).

In this study we evaluated X-ray irradiation as a sterilization method for decontaminating *A*. *fumigatus*, *A*. *niger*, *A*. *flavus*, and *A*. *terreus* in dried cannabis flowers. Gamma irradiation and e-beam irradiation both require large, specialized facilities that we did not have access to, and as a result, we could not test these methods of ionizing radiation.

## Materials and methods

### Sample preparation for microbial analysis

Dried cannabis flowers (trimmed buds) were grown in California and provided by the Bureau of Cannabis Control. Cannabis flower samples were weighed into sterile Whirlpak Filter bags (Nasco Sampling LLC) (1.00±0.05 g/bag). Samples were pre-treated with pre-determined dose of 2.5 kGy of X-ray irradiation to inactivate any naturally occurring *Aspergillus*. X-ray irradiation was applied using a Rad Source 420M (Rad Source Technologies Inc., Buford, GA) which was operated according to manufacturer’s instructions. Samples were then spiked with *Aspergillus* spores.

### Strain preparation

The *Aspergillus* strains used in this study (Table 1) were grown for 2 to 5 days at 30°C on potato dextrose agar (PDA). Spores were harvested from PDA plates by washing with 10 mL sterile 0.05% Tween 20 in water and quantified by hemocytometer cell counting. Spore suspensions were then normalized and serially diluted to the desired concentrations.

**Table 1:** *Aspergillus* strains used in this study.

**Organism**	**Strain**
*Aspergillus niger*	ATCC 16888
*Aspergillus fumigatus*	ATCC 1022
*Aspergillus flavus*	ATCC 16870
*Aspergillus terreus*	ATCC 20541

### *Aspergillus* enrichment and detection by real-time PCR

After X-ray treatments, potato dextrose broth containing 0.05% chloramphenicol was added to each cannabis sample to make a 1:25 dilution (1 g sample in 24 mL media). Samples were then incubated at 30°C for 48 ± 4 hours. After incubation, 5 mL of sample enrichment was removed for PCR analysis.

The 5 mL sample enrichments were centrifuged at 5000 rpm for 2 minutes and the supernatant was discarded. Pellets were resuspended in 500 μL lysis buffer [41] (400 mM Tris-HCl [pH 8.0], 60 mM EDTA [pH 8.0], 150 mM NaCl, 1% sodium dodecyl sulfate) and added to a screw-cap microcentrifuge tube with sterile glass beads (Sigma G1277, 212–300 μm) filled to approximately the 0.1 mL line.

Bead-beating was performed at 6m/s for 1 minute on a FastPrep24-5G (M.P. Biomedicals, Santa Ana, CA), after which samples were placed on ice for 1 minute. DNA extraction continued as described by Liu *et al*. [41] from step (ii) with minor modifications. DNA pellets were resuspended in 50 μL sterile distilled water, and 2 μL was used for real-time PCR.

PCR reactions were performed using a QuantStudio 5 (Applied Biosystems, Foster City, CA) in a 20 μL reaction volume containing TaqPath ProAmp Multiplex Master Mix (Applied Biosystems Cat# A30868), 1 μL Thermo Fisher *Aspergillus* multiplex v2 (Custom TaqMan Aspergillus Kit v2, Cat# A43213C), and 2 μL DNA. The following cycling conditions were used: 95°C for 5 minutes, followed by 40 cycles of 95°C for 5 seconds and 63°C for 30 seconds. Reporter dyes were used with the following targets: FAM (*fumigatus*), VIC (*flavus*), ABY (*terreus*), and JUN (*niger*). Mustang Purple was used as a passive reference dye.

Real-time PCR results were analyzed using QuantStudio Design and Analysis Software v1.5.1. Thresholds for each target were set as follows: *niger* (0.2), *fumigatus* (0.2), *flavus* (0.8), *terreus* (0.8).

Viability of *Aspergillus* in the cannabis sample enrichments was determined by plating onto PDA using a 10 μL loop and incubating for up to 5 days at 30°C. Plates were examined for typical *Aspergillus* growth for the appropriate species.

### X-ray irradiation and *Aspergillus* viability

To determine the dosage required to inactivate *A*. *niger*, *A*. *fumigatus*, *A*. *flavus*, and *A*. *terreus* spores, large amounts of spores (>10^6^), from each *Aspergillus* species, were spiked into 1 gram of dried cannabis flower in Whirlpak bags. The spiked cannabis samples were then exposed to 2.0, 2.5, or 5.0 kGy of X-ray irradiation. Following irradiation, all samples were tested and viability confirmed as described above.

In a second assay, 1 gram cannabis flower samples were inoculated with low (10^2^ spores), medium (10^3^ spores), or high (10^4^ spores) amounts of *A*. *niger*, *A*. *fumigatus*, *A*. *flavus*, or *A*. *terreus* spores. “Treated” samples were exposed to 2.5 kGy of X-ray irradiation. Similarly, inoculated control samples were prepared and received no x-ray exposure and served as “untreated” samples. All samples were tested in triplicate.

In a third assay, for a larger scale experiment, two 100 g cannabis flower samples were spiked with 10^6^ *A*. *niger* spores/100 g flower. One sample was then exposed to 2.5 kGy of X-ray irradiation and the other left untreated. Two 10 g subsamples were randomly taken from each sample (including an additional 100 g matrix control) and tested as described above.

### Sample preparation for chemical analysis

Dried cannabis flowers (trimmed buds) were ground and homogenized using GenoGrinder from SPEX SamplePrep (Metuchen, NJ), and seven bags each containing 3 grams of ground flowers were prepared for X-ray irradiation treatment. Ground cannabis flower samples were treated at six different X-ray irradiation dosage level (0.5, 1.0, 1.5, 2.0, 2.5 and 5.0 kGy). A triplicate of 200 mg samples was weighed out from each X-ray treated samples as well as the untreated sample. The samples were extracted with 40 mL extraction solvents (80 acetonitrile: 20methanol, v/v), sonicated for 30 min, followed by centrifugation at 3900 rpm for 15 min. The supernatant was filtered through a 0.25 μm PTFE filter and further diluted 200-fold and 400-fold, respectively before loading onto the instrument for qualitative and quantitative analyses. A seven-point calibration curve from 20 ppb to 2000 ppb was used to determine the concentration of the cannabinoids in the samples. Isotopically labeled THC-d_3_ was added to each calibration standard and all samples to serve as internal standard. Quality control samples were included in the batch to ensure accuracy and precision of the method.

### Cannabinoid quantitative analysis by UHPLC-MS/MS

The quantitative analysis of eight different cannabinoids (namely, CBDA, CBD, THCA, delta9-THC, CBN, CBG, THCV and CBC) were performed on a SCIEX Exion UHPLC system coupled with a triple quadrupole mass spectrometer (4500) with an ESI source (Framingham, MA). SCIEX Analyst software (1.7.0) was used to control the instrument and collect data. All eight cannabinoids reference standards CBDA, CBD, THCA, delta9-THC, CBN, CBG, THCV and CBC and the internal standards (THC-d3) were purchased from Cerilliant (Round Rock, TX). LCMS grade Methanol, acetonitrile, water and formic acid were purchased from Fisher Scientific.

The LC column used for this method was a Cortecs UPLC C18 column (100mm× 2.1mm I.D., 1.6 μm) from Waters (Milford, MA). The column oven temperature was set at 35°C. The mobile phases consisted of (A) 0.05% formic acid in water and (B) 0.05% formic acid in acetonitrile. The following gradient was used to achieve chromatographic separation of the cannabinoids at a flow rate of 0.25 ml/min: 0–8min, 70 to 80% B; 8–10 min, 80 to 100% B, followed by a 4-min washing procedure with 100% B and a re-equilibration period of 4 min with initial conditions. The total run time was 18 min for each sample. The injection volume was 2 μL. The autosampler was maintained at 15°C. For the MS/MS parameters, both positive and negative mode were used for detecting different cannabinoids: CBD, delta9-THC, CBN, CBG, THCV and CBC were detected using positive mode and the acidic cannabinoids CBDA and THCA were detected using negative mode. Ion spray voltage was set to 4500 for the positive mode and -4500 for the negative mode. Curtain gas was set to 40 psi. Source Temperature (TEM) was 500°C. Ion source gas 1 and 2 (GS1 and GS2) were both set at 50 psi. Two different ion transitions were used for each cannabinoid.

### Cannabinoids and terpenes qualitative analysis by GC-MS

The gas chromatography mass spectrometry (GC-MS) screen method uses a full scan mode in MS to tentatively identify known and unknown/non-targeted chemical substances in a sample based on a match to an established mass spectral library. This method was used to qualitatively determine cannabinoids and terpenes profile changes before and after X-ray irradiation. One mL 200-fold diluted samples were spiked with an internal standard mix [Triphenylphosphate and Phenanthrene-d_10_, (Sigma-Aldrich, Saint Louis, MO)], before being injected on Agilent GC7890B coupled with MS5977B (Agilent, Santa Clara, CA). Quality control samples were included in the sample batch.

The injection volume was 1 μL and the splitless mode was used at the GC injection port. Chromatographic separation was achieved in a 30 min run time using a DB-5MS column (30 m x 0.25 mm x 0.25 μm, Agilent) with 1 mL/min helium flow. The oven temperature program was set at 60°C at 1 min, followed by a 12°C/min ramp to 320°C and hold for 7.3 min. The transfer line temperature was set at 280°C, the ion source temperature at 250°C, and EI ionization energy at 70eV. Mass spectral data was acquired in the scan mode from 25 to 550 m/z at a speed of 2.8 scan/s. Tentative compound identifications are based on a comparison of electron impact mass spectra with the Wiley11/NIST 2017 Mass Spectral libraries and Cayman Spectral library. The match criteria from the compound mass spectra to the database must have a fit of greater than 90% match ratio and visually verified by the analyst. Major cannabinoids and terpenes detected in samples were confirmed with the standards purchased from Sigma-Aldrich, Cerilliant, and Emerald Scientific (San Luis Obispo, CA).

## Results

### X-ray exposure and *Aspergillus* viability

To determine the minimal X-ray dosage required for inactivation of *Aspergillus*, we tested samples spiked with one of the four *Aspergillus* spp. (Table 1), followed by X-ray irradiation at 2.0, 2.5, or 5.0 kGy and subsequent plating onto PDA for fungal growth assessment. After 2.0 kGy of X-ray irradiation, some growth was detected in samples spiked with *A*. *fumigatus* and *A*. *flavus*. No *Aspergillus* growth on PDA plates was observed after 2.5 and 5.0 kGy of X-ray irradiation; however, bacterial growth was detected in samples irradiated with 2.5 kGy. 2.5 kGy was determined to be the lowest amount of X-ray exposure that is sufficient to inactivate the viability of *Aspergillus* spores, see Table 2.

**Table 2:** PCR detection and viability of various *Aspergillus* spp. after X-ray irradiation.

	2.0 kGy	2.5 kGy	5.0 kGy
*Aspergillus niger*	NG	NG	NG
*Aspergillus fumigatus*	G	NG	NG
*Aspergillus flavus*	G	NG	NG
*Aspergillus terreus*	NG	NG	NG

G: *Aspergillus* growth observed on PDA plates

NG: No *Aspergillus* growth on PDA plates

### X-ray irradiation with quantitative spike levels

No viability was observed for any of the *Aspergillus* spp. in the samples exposed to 2.5 kGy, regardless of inoculation level. Without X-ray irradiation, *Aspergillus* growth was observed even at the lowest spike levels. At medium and high spike levels, DNA from *Aspergillus* spores were detectable by PCR, even after X-ray exposure. In samples without X-ray exposure irradiation, respective *Aspergillus spp*. were detected by PCR regardless of spike level, see Tables 3–6. Irradiated medium and high spike samples resulted in detectable Ct values although with later cycle thresholds than comparable untreated samples. A sample was considered “positive”, DNA detected, for an *Aspergillus* target if fluorescent curves crossed the cycle threshold (Ct) line ≤40. A Ct of zero means that the fluorescent curve did not cross the cycle threshold.

**Table 3:** PCR detection and viability of *A*. *niger* at three spike levels, treated or untreated with 2.5 kGy of X-ray irradiation.

	Untreated		X-ray Treated	
*A*. *niger*	Growth on PDA	Ct	Growth on PDA	Ct
Low^1^ spike 1	G	22.77	NG	0
Low spike 2	NG	19.52	NG	0
Low spike 3	G	16.07	NG	0
Medium^2^ spike 1	G	16.27	NG	35.88
Medium spike 2	G	16.53	NG	35.29
Medium spike 3	G	15.03	NG	34.47
High^3^ spike 1	G	16.03	NG	29.35
High spike 2	G	15.69	NG	31.74
High spike 3	G	15.46	NG	31.08
Matrix no spike 1	NG	0	NG	0
Matrix no spike 2	NG	0	NG	0
Matrix no spike 3	NG	0	NG	0

^1^ Low spike: 10^2^ spores/1 g flower

^2^Medium spike: 10^3^ spores/1 g flower

^3^High spike: 10^4^ spores/1 g flower

G: *Aspergillus* growth observed on PDA plates

NG: No *Aspergillus* growth observed on PDA plates

**Table 4:** PCR detection and viability of *A*. *fumigatus* at three spike levels, treated or untreated with 2.5 kGy of X-ray irradiation.

	Untreated		X-ray Treated	
*A*. *fumigatus*	Growth on PDA	Ct	Growth on PDA	Ct
Low^1^ spike 1	G	29.55	NG	0
Low spike 2	G	37.02	NG	0
Low spike 3	G	32.33	NG	38.78
Medium^2^ spike 1	G	27.21	NG	37.5
Medium spike 2	G	30.97	NG	37.18
Medium spike 3	G	29.13	NG	39.77
High^3^ spike 1	G	33.91	NG	34.63
High spike 2	G	25.92	NG	37.15
High spike 3	G	28.46	NG	35.63
Matrix no spike 1	NG	0	NG	0
Matrix no spike 2	NG	0	NG	0
Matrix no spike 3	NG	0	NG	0

^1^ Low spike: 10^2^ spores/1 g flower

^2^Medium spike: 10^3^ spores/1 g flower

^3^High spike: 10^4^ spores/1 g flower

G: *Aspergillus* growth observed on PDA plates

NG: No *Aspergillus* growth observed on PDA plates

**Table 5:** PCR detection and viability of *A*. *flavus* at various spike levels, treated or untreated with 2.5 kGy of X-ray irradiation.

	Untreated		X-ray Treated	
*A*. *flavus*	Growth on PDA	Ct	Growth on PDA	Ct
Low^1^ spike 1	NG	33.34	NG	0
Low spike 2	G	24.1	NG	0
Low spike 3	G	33.8	NG	0
Medium^2^ spike 1	G	17.2	NG	36.97
Medium spike 2	G	19.3	NG	36.13
Medium spike 3	G	19.74	NG	37.93
High^3^ spike 1	G	18.41	NG	33.92
High spike 2	G	17.15	NG	33.82
High spike 3	G	17.86	NG	31.86
Matrix no spike 1	NG	0	NG	0
Matrix no spike 2	NG	0	NG	0
Matrix no spike 3	NG	0	NG	0

^1^ Low spike: 10^2^ spores/1 g flower

^2^Medium spike: 10^3^ spores/1 g flower

^3^High spike: 10^4^ spores/1 g flower

G: *Aspergillus* growth observed on PDA plates

NG: No *Aspergillus* growth observed on PDA plates

**Table 6:** PCR detection and viability of *A*. *terreus* at various spike levels, treated or untreated with 2.5 kGy of X-ray irradiation.

	Untreated		X-ray Treated	
*A*. *terreus*	Growth on PDA	Ct	Growth on PDA	Ct
Low^1^ spike 1	G	28.87	NG	0
Low spike 2	NG	31.59	NG	0
Low spike 3	NG	32.12	NG	0
Medium^2^ spike 1	G	24.22	NG	0
Medium spike 2	NG	23.5	NG	35.89
Medium spike 3	G	23.1	NG	37.33
High^3^ spike 1	G	20.53	NG	32.31
High spike 2	G	21.25	NG	34.59
High spike 3	G	23.54	NG	34.74
Matrix no spike 1	NG	0	NG	0
Matrix no spike 2	NG	0	NG	0
Matrix no spike 3	NG	0	NG	0

^1^ Low spike: 10^2^ spores/1 g flower

^2^Medium spike: 10^3^ spores/1 g flower

^3^High spike: 10^4^ spores/1 g flower

G: *Aspergillus* growth observed on PDA plates

NG: No *Aspergillus* growth observed on PDA plates

Similar results were observed in the 100 g scale-up experiment: each 10 g subsample exposed to 2.5 kGy of X-rays showed no *A*. *niger* viability, while those without X-ray exposure had *Aspergillus* growth, See Table 7.

**Table 7:** 100 g cannabis flower scale-up experiment.

Sample	Subsample	X-ray exposure	PCR	Growth on PDA
1	A	2.5 kGy	ND	NG
	B	2.5 kGy	ND	NG
2	A	0 kGy	D	G
	B	0 kGy	D	G
Matrix control	A	0 kGy	ND	NG
	B	0 kGy	ND	NG

ND: *Aspergillus* DNA Not Detected

D: *Aspergillus* DNA Detected

G: *Aspergillus* growth observed on PDA plates

NG: No *Aspergillus* growth observed on PDA plates

### Cannabinoids profile by LC-MSMS

A total of twenty-one samples were used in this study—three untreated cannabis flower samples and three replicate samples from each of the six X-ray dosage levels, respectively.

Table 8 shows the average of the cannabinoid concentrations of the three replicate samples before and after the X-ray irradiation treatment. The top two most abundant cannabinoids in the tested flower samples were THCA and delta9-THC, respectively. The slight differences in concentration of THCA/delta9-THC at different levels are mainly due to the natural inhomogeneity of the cannabis flower sample and the measurement uncertainty for all analytical methods. A trend test across different treatment groups was performed on STATA version 17.0 (StataCorp) for potential cannabinoids profile changes after X-ray irradiation. We set the level of significance as *p* < 0.05. No significant trends were observed for THCA and delta9-THC concentrations.

**Table 8:** Cannabinoid concentration of flower samples before and after X-ray irradiation treatment at different dosage levels.

	CBDA	THCV	CBD	CBG	CBN	THC	THCA	CBC
X-ray dose	mg/g	mg/g	mg/g	mg/g	mg/g	mg/g	mg/g	mg/g
0.0 (untreated)	0.623	0.298	ND	1.10	0.558	38.9	140	0.959
0.5	0.654	0.311	ND	1.20	0.649	42.5	154	1.12
1.0	0.670	0.313	ND	1.21	0.663	42.6	154	1.12
1.5	0.680	0.307	ND	1.21	0.688	42.9	157	1.16
2.0	0.665	0.313	ND	1.19	0.694	41.5	153	1.11
2.5	0.632	0.282	ND	1.06	0.662	37.6	140	1.01
5.0	0.618	0.276	ND	1.03	0.715	36.3	138	0.970
Average	0.649	0.300	ND	1.14	0.661	40.3	148	1.06
RSD%	3.78	5.07	ND	6.47	7.70	6.67	5.59	7.82

RSD: Relative Standard Deviation

ND: Not Detected

Concentrations for CBDA, THCV, CBG and CBC are less than the reporting limit (1.6mg/g or 0.16%) but mostly unchanged for each X-ray dosage level. There was no CBD identified in the tested cannabis flower samples.

**Fig 1:**
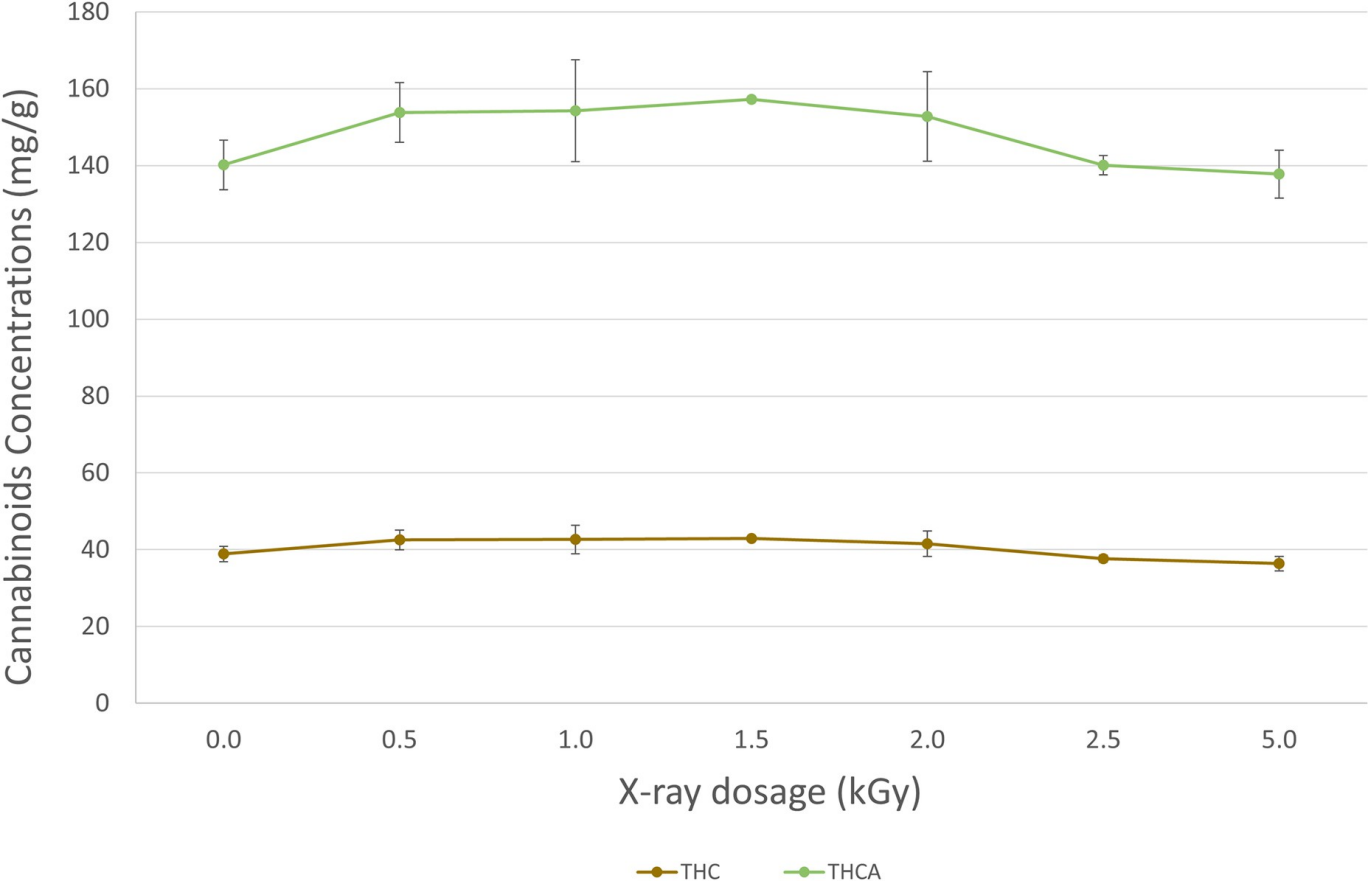
Cannabinoid concentrations in flower samples of untreated and treated at different X-ray irradiation dosage levels.

The temperature in the X-ray chamber was monitored during the X-ray irradiation process, which was slightly elevated to around 28°C compared to the 23°C at the beginning. This slight elevation of temperature did not seem to cause accelerated decarboxylation of the THCA to THC nor degradation of the THC to CBN in the sample, as there is no trend observed for changes in the THCA and THC concentrations. Fig 1 shows the average THCA and delta9-THC concentrations of the flower samples treated at different dosage levels, with error bars calculated using standard deviation of the three replicates for each level. It is reasonable to conclude that the X-ray irradiation has minimal impact on the THCA and delta9-THC of the cannabis flower samples.

### Qualitative cannabinoids profile by GC-MS

To confirm the findings of cannabinoids profile by LC-MSMS, GC-MS screen method was also used in this study. Four cannabis flower samples ‒ one untreated and three treated with 1.0, 2.5 and 5.0 kGy dose levels were analyzed to determine if cannabinoid and terpene profiles had changed after X-ray irradiation. A second set of samples were used to confirm the findings. The peak area percentages of the compounds generated from GC-MS analysis were used for the qualitative analysis. Peak area percentage of each peak roughly represents the composition and amount of analyte (e.g., Delta9-THC) observed in the sample.

**Fig 2:**
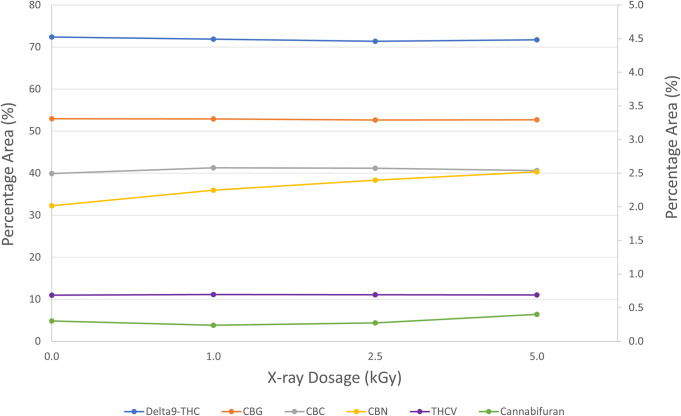
Qualitative cannabinoids profile changes in flower samples of untreated and treated at different X-ray irradiation dosage levels. Primary axis for delta9-THC, secondary axis for CBG, CBC, CBN, THCV, and cannabifuran. % Peak Area = area percentage of each peak or compound found in instrument analysis. It only roughly represents composition and amounts present in the sample.

The major cannabinoids found in flower samples were Delta9-THC (~70% peak area), CBG (~ 3% peak area), CBC (2.5% peak area), CBN (2.5% peak area), THCV (0.7% peak area), and cannabifuran (0.3% peak area) (Fig 2). Due to high temperature on heated GC-MS injection port, THCA, CBDA and other acid form of cannabinoids were not observed by GC-MS as they were mostly decarboxylated to the neutral cannabinoids such as THC and CBD. We did not observe any changes for Delta9-THC, CBG, CBC, and THCV. There was a slight increase in CBN amount as the treatment doses increased. CBN is an oxidation and degradation byproduct of the Delta9-THC and the slight increase may be due to slight increase in temperatures at higher levels of the X-ray treatment. There was also a slight increase in cannabifuran amount at the highest treatment dose level (5.0 kGy). The qualitative results observed were not tested for statistical significance. The cannabinoid profile by GC-MS screen method is consistent with the results found using LC-MSMS quantitative analysis.

### Qualitative terpenes profile by GC-MS

**Fig 3:**
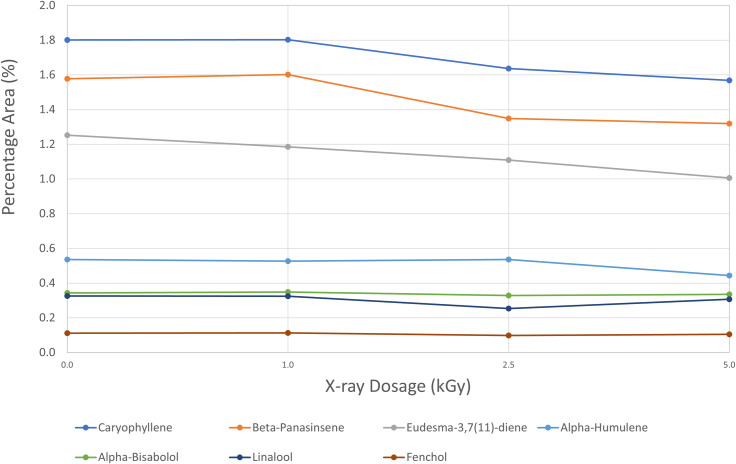
Qualitative terpenes profile changes in flower samples of untreated and treated at different X-ray irradiation dosage levels. % Peak Area = area percentage of each peak or compound found in instrument analysis. It only roughly represents composition and amounts present in the sample.

The major terpenes found in flower samples were Caryophyllene (1.8% peak area), Beta-Panasinsene (1.6% peak area), Eudesma-3,7(11)-diene (1.2% peak area), Alpha-Humulene (0.5% peak area), Alpha-Bisabolol (0.3% peak area), Linalool (0.3% peak area), and Fenchol (0.1% peak area) (Fig 3). We didn’t observe any changes for Alpha-Bisabolol and Fenchol. It seemed that there was a slight downward trend for Caryophyllene, Beta-Panasinsene, and Eudesma-3,7(11)-diene. Alpha-Humulene had a lower level at the highest treatment level (5.0 kGy). These changes were not tested for statistical significance. Terpenes are volatile chemical compounds and these small changes may reflect the slight increase in temperatures at higher levels of the X-ray treatment.

## Discussion

In this study we examined whether X-ray irradiation is a feasible method of inactivating *Aspergillus* in cannabis flower contaminated with four pathogenic species of *Aspergillus* (*A*. *niger*, *A*. *terreus*, *A*. *fumigatus* or *A*. *flavus*). We demonstrated that X-ray irradiation at 2.5KGy of cannabis flower contaminated with any of the four pathogenic *Aspergillus* species can render the pathogen non-viable to at least a spiking level of 10^4^ CFUs/gram. We also showed that the genetic material of killed organisms remains in the irradiated product and is detectable via molecular based methods such as qPCR. This is a very important finding and should be taken into consideration when deciding what detection method to use to assess the success of the decontamination process. We recommend using a culture-based method in addition to molecular-based methods to test the decontaminated product and thus determine the true clearance of the live organism.

We also showed that X-ray treatment of *Aspergillus* contaminated cannabis flower at 2.5 kGy has minimal effects on THCA, Delta9-THC and terpene concentrations. However, our experiment was conducted under well controlled laboratory conditions and does not reflect how cannabis manufacturers may conduct their decontamination procedures. We recommend that the X-ray remediated samples be tested as a new product batch, in order to meet the testing regulation requirements.

Taken together, our findings suggest that the use of X-ray technology may be a useful method to inactivate *Aspergillus* in contaminated cannabis flower. Further work is needed to assess whether X-ray irradiation has any deleterious effect on the cannabis flower’s genetic, chemical, or phenotypic profile and thus may pose a safety issue to the consumers.
